# Supplementary material for: Nitrogen Signaling Genes and SOC1 Determine the Flowering Time in a Reciprocal Negative Feedback Loop in Chinese Cabbage (Brassica rapa L.) Based on CRISPR/Cas9-Mediated Mutagenesis of Multiple BrSOC1 Homologs
Source: Int J Mol Sci. 2021 Apr 28;22(9):4631. doi: 10.3390/ijms22094631 (PMC8124421; doi:10.3390/ijms22094631)
Supplement: Supplementary file 1 [file ijms-22-04631-s001.zip › ijms-1174205 supplementary.v7.pdf]

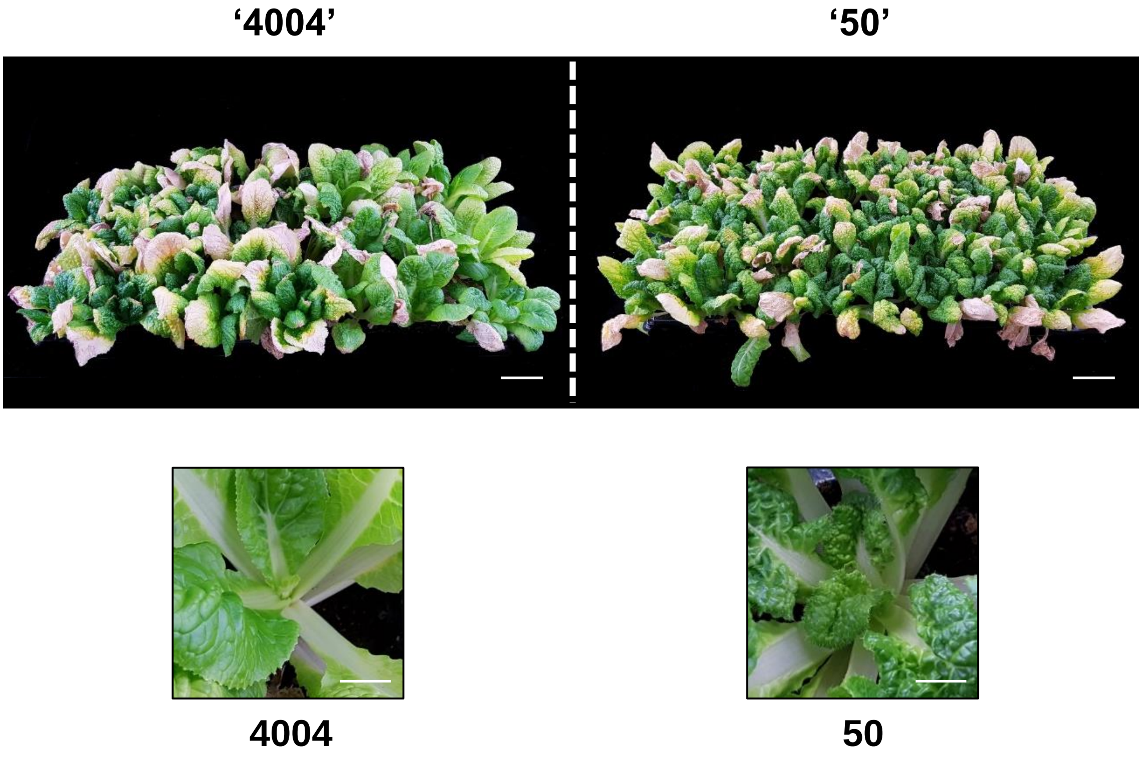

Figure S1. Bolting phenotypes of two Chinese cabbage inbred lines without vernalization. Germinated seedlings were grown in a growth room (23 °C, 16 h light/8 h dark) for 12 weeks without switching to vernalization conditions. Upper pictures, scale bars = 5 cm; lower pictures, scale bars = 1 cm.

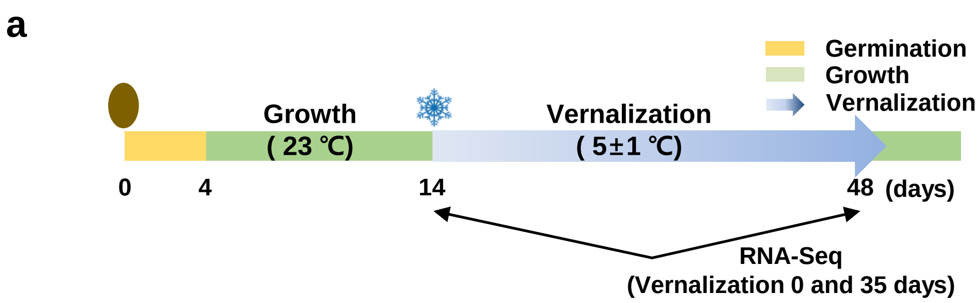

Inbred lines	'4004'		'50'	
Vernalization	0d	35d	0d	35d
Samples	1st, 2nd, 3rd	1st, 2nd, 3rd	1st, 2nd, 3rd	1st, 2nd, 3rd

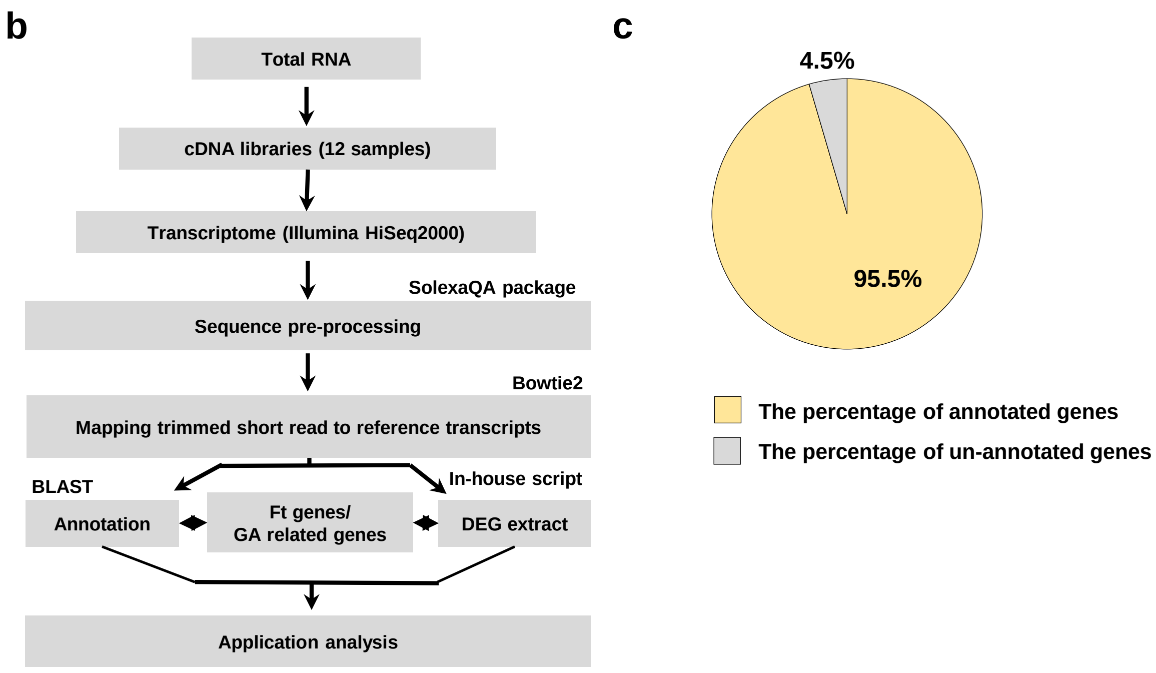

Figure S2. Experimental design for RNA-seq analysis. **(a, b)** Samples of line '4004' and line '50' plants were subjected to vernalization for 0 and 35 days and used for RNA-seq analysis. 0d, 0 days vernalization; 35d, 35 days vernalization; 1st, 2nd, 3rd, independent biological triplicates. Schematic presentation of the workflow and algorithm used to detect DEGs and flowering-time genes. **(c)** Annotation statistics analysis. Genes were analyzed using the B. rapa functional annotation set; Brapa_sequence_v1.5_CDS.

a

0D Ver, up-regulated in 50

Go IDs	Go terms	Gene counts	Ft genes
GO:0006807	Nitrogen compound meta bolic process	449	0
GO:0044271	Cellular nitrogen compou nd biosynthetic process	339	0
GO:0019740	Nitrogen utilization	5	0
GO:0042126	Nitrate metabolic process	6	0
GO:2001057	Reactive nitrogen specie s metabolic process	6	0
GO:1901698	Response to nitrogen co mpound	46	0
GO:1905392	Plant organ morphogene sis	79	0
GO:0048438	Floral whorl development	43	0
GO:0009791	Post-embryonic develop ment	130	0
GO:0048437	Floral organ development	43	0
GO:0061458	Reproductive system dev elopment	86	0

35D Ver, up-regulated in 50

Go IDs	Go terms	Gene counts	Ft genes
GO:0042126	Nitrate metabolic process	5	0
GO:2001057	Reactive nitrogen species m etabolic process	5	0
GO:0006809	Nitric oxide biosynthetic pro cess	3	0
GO:0046209	Nitric oxide metabolic proce ss	3	0
GO:0006807	Nitrogen compound metaboli c process	110	0
GO:0008940	Nitrate reductase activity	3	0
GO:0098809	Nitrite reductase activity	2	0
GO:0048509	Regulation of meristem deve lopment	7	0

b

0D Ver, up-regulated in 4004

Go IDs	Go terms	Gene counts	Ft genes
GO:0006950	Response to stress	284	5
GO:0050896	Response to stimulus	384	6
GO:0009628	Response to abiotic stimulus	192	5
GO:0009725	Response to hormone	157	4
GO:0009409	Response to cold	62	1
GO:0048583	Regulation of response to sti mulus	74	2
GO:0009631	Cold acclimation	7	0
GO:0048281	Inflorescence morphogenesis	4	0
GO:0010150	Leaf senescence	13	0
GO:0090693	Plant organ senescence	13	0
GO:0010229	Inflorescence development	4	0

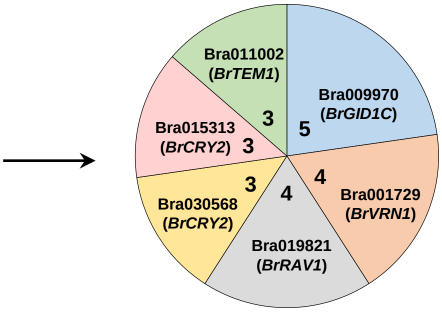

Figure S3. Identification of flowering time (Ft) genes based on gene ontology (GO) terms. **(a)** Detection of Ft genes among up-regulated DEGs in line '4004', 0D Ver conditions (left Table); 35D Ver conditions (right Table). **(b)** Detection of Ft genes among up-regulated DEGs in line '50' under 0D Ver conditions.

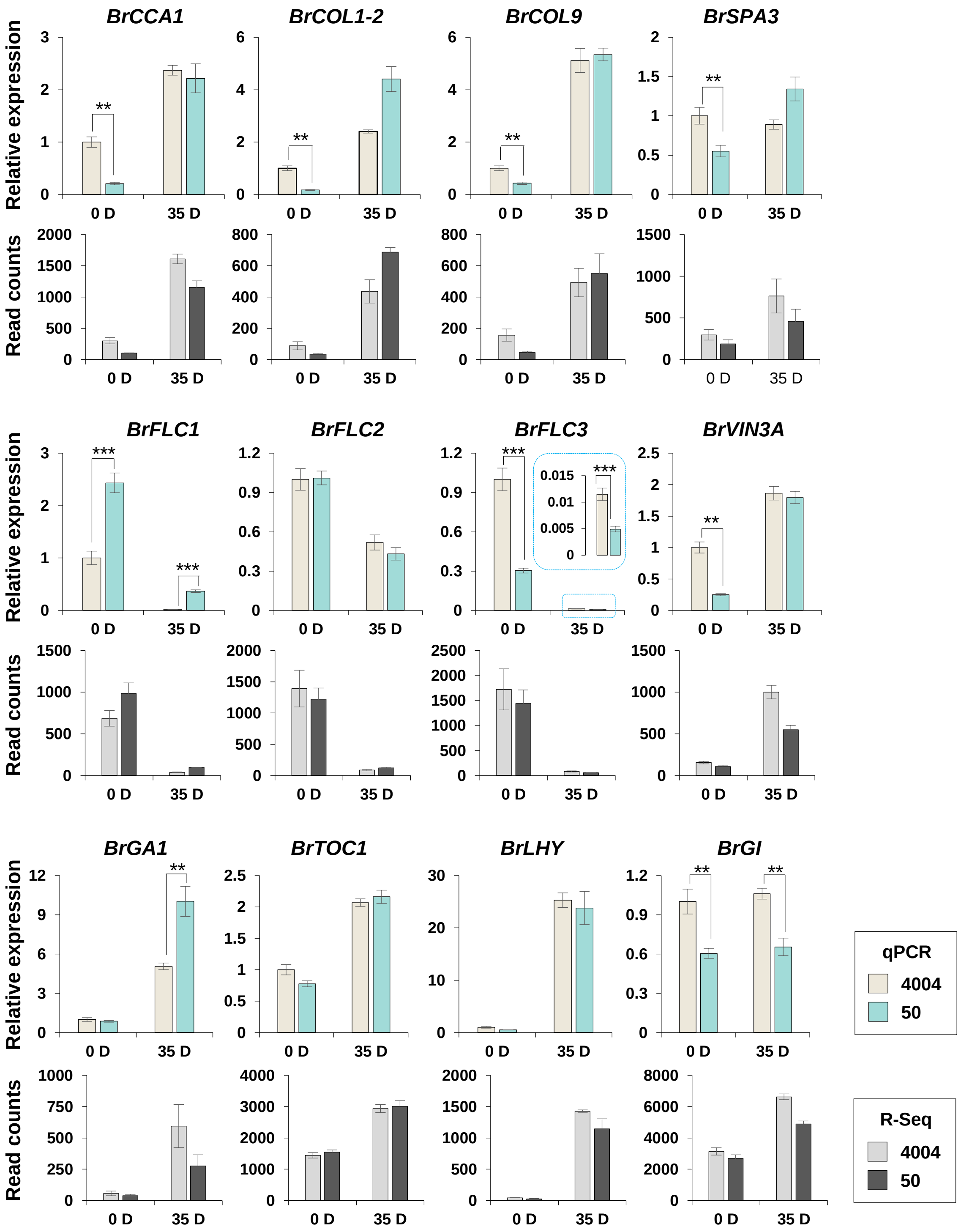

Figure S4. Results of qPCR and RNA-seq analysis of flowering time (Ft)-related genes in the inbred lines '4004' and '50' grown with or without vernalization. This analysis was done in the same leaf samples (+/- Vernalization) used for RNAseq. qPCR values were normalized against the corresponding level of internal control gene actin (BrACT2). For each gene in the qPCR analysis, the expression level from '4004' on day 0 was defined as "1". Error bars represent ± SE of three replicates (**p < 0.05, *** p < 0.01; Student's t-test).

```
BrSOC1-1    Bra004928     TCTCTGATCATCTTCTCTC-CTAAGGCAAAACTTTAT
            Indel (+ C)   TCTCTGATCATCTTCTCTCCCTAAGGCAAAACTTTAT
                            S  L  I  I  F  S   P  K  A  K  L  Y
                            S  L  I  I  F  S   P  *

BrSOC1-2    Bra000393     TCTCTGATCATCTTCTCTC-CTAAGGGAAAACTTTAT
            Indel (- C)   TCTCTGATCATCTTCTCT--CTAAGGGAAAACTTTAT
            Indel (+ A)   TCTCTGATCATCTTCTCTCACTAAGGGAAAACTTTAT
                            S  L  I  I  F  S   P  K  G  K  L  Y
                            S  L  I  I  F  S   L  R  E  N  F
                            S  L  I  I  F  S   H  *

BrSOC1-3    Bra039324     TCTCTGATCATCTTCTCTC-CTAAGGGGAAAACTTTA
            Indel (+ A)   TCTCTGATCATCTTCTCTCACTAAGGGGAAAACTTTA
                            S  L  I  I  F  S   P  K  G  K  T  L
                            S  L  I  I  F  S   H  *
```

Figure S5. Multiple sequence alignment of insertion/deletion (indel) variants of three BrSOC1 homologs.

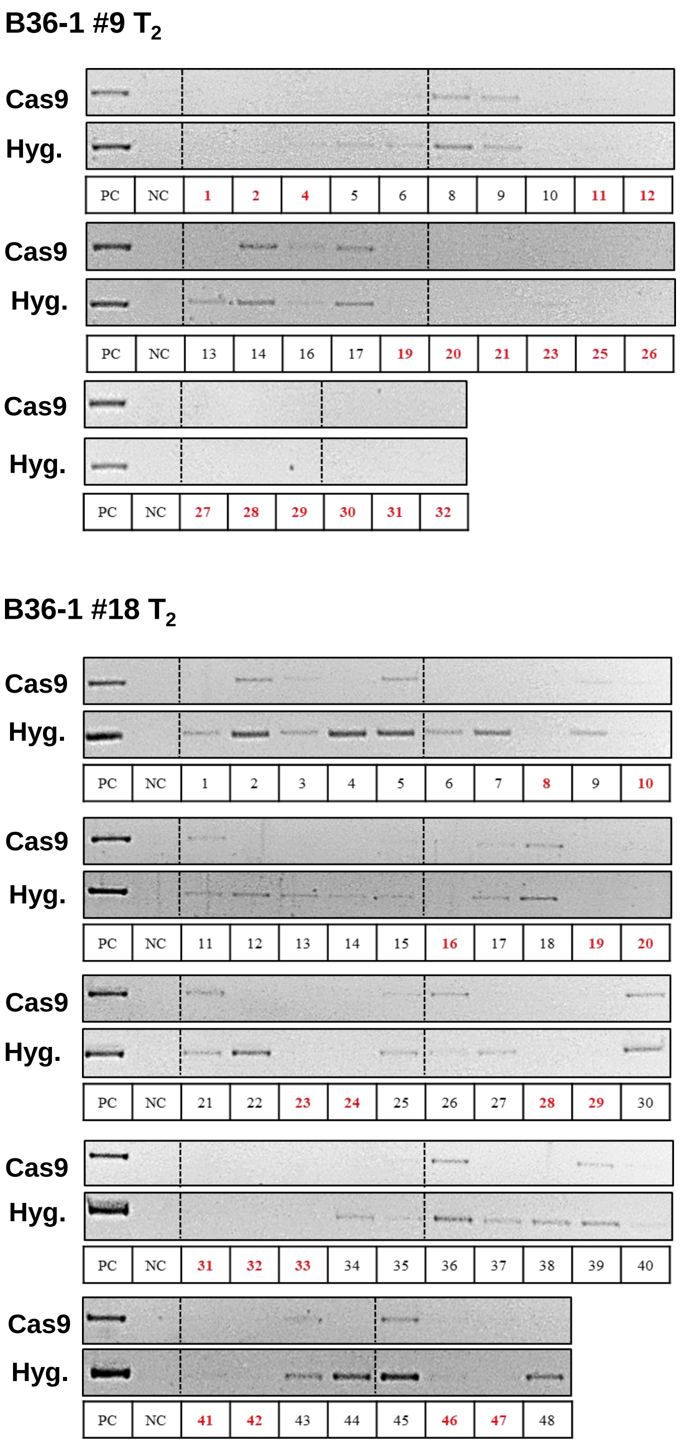

Figure S6. Cas9 and Hygromycin (Hyg.) PCR analysis in B36-1 T_2 plants. PC, positive control (pHAtC vector); NC, negative control (‘20’ WT). Red numbers mean deletion of Cas9 and Hyg.

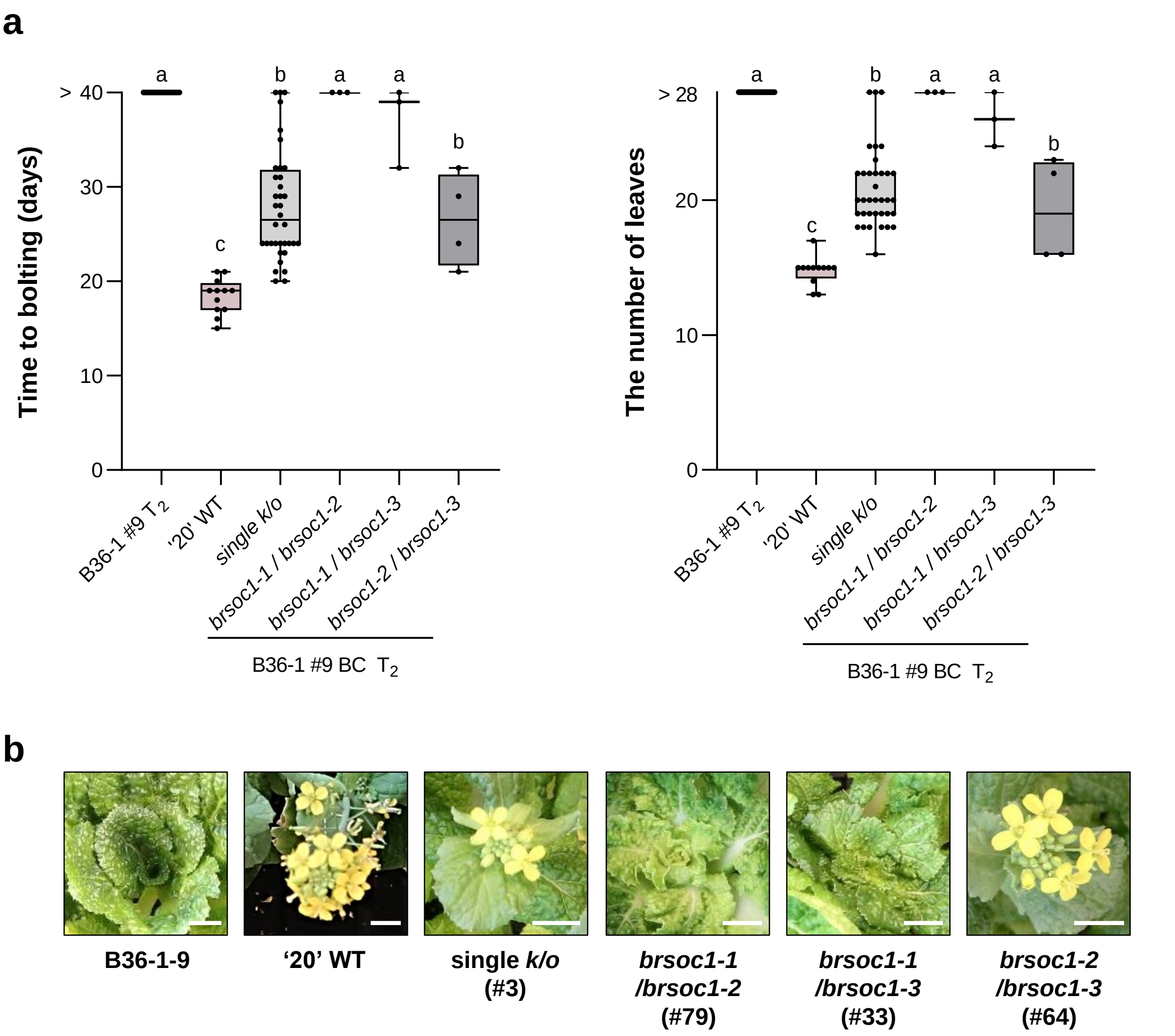

Figure S7. Backcrossing (BC) of *brsoc1s* gene-edited Chinese cabbage. **(a)** Statistical analysis of the number of days to bolting after vernalization, and the number of leaves in the six genotypic groups. The leaves were counted when the plants started bolting. Statistically significant differences are indicated with different letters ($p < 0.05$, one-way ANOVA followed by Tukey post-hoc test, each dot indicates the number of plants (n); B36-1 #9 T_2 = 32, '20' WT = 12, single *k/o* (*brsoc1-1*, *brsoc1-2* or *brsoc1-3*) = 36, double *k/o*; *brsoc1-1/brsoc1-2* = 3, *brsoc1-1/brsoc1-3* = 3, *brsoc1-2/brsoc1-3* = 4). **(b)** Bolting phenotype between B36-1-9, WT '20', and B36-1 BC T_2 plants (selfing after backcrossing). Seedlings were vernalized for 35 days at 5 ± 1 °C using the 16 h light / 8 h dark photoperiod and then transferred to a growth room at 23 °C for more than 40 days. Scale bars = 1 cm.

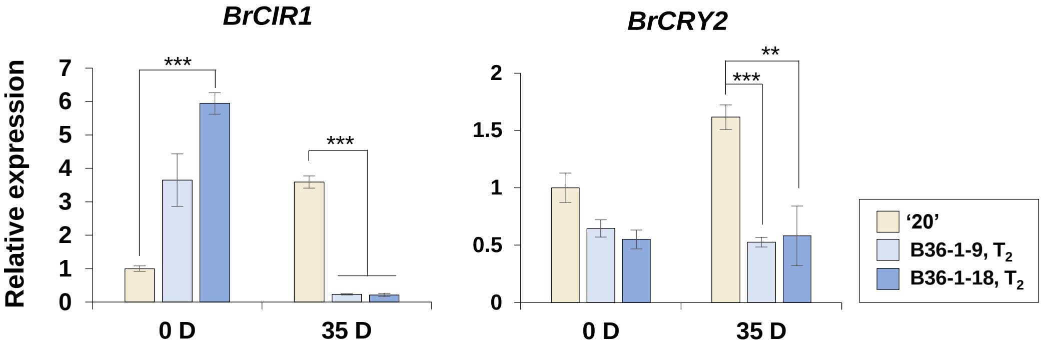

Figure S8. Quantitative PCR analysis in BrSOC1s-edited Chinese cabbage plants grown with or without vernalization. This analysis was done in the same leaf samples (+/- Vernalization) used for Figure 7. Gene expression after normalization based on actin (*BrACT2*) transcript abundance. The expression level of genes in '20' WT on day 0 (no Vernalization) was defined as "1". Error bars represent SE of three replicates (**$p < 0.05$, ***$p < 0.01$; Student's *t*-test).
